# High burden and seasonal variation of paediatric scabies and pyoderma prevalence in The Gambia: A cross-sectional study

**DOI:** 10.1371/journal.pntd.0007801

**Published:** 2019-10-14

**Authors:** Edwin P. Armitage, Elina Senghore, Saffiatou Darboe, Momodou Barry, Janko Camara, Sulayman Bah, Michael Marks, Carla Cerami, Anna Roca, Martin Antonio, Claire E. Turner, Thushan I. de Silva

**Affiliations:** 1 Medical Research Council Unit The Gambia at The London School of Hygiene & Tropical Medicine, Banjul, The Gambia; 2 Department of Clinical Research, Faculty of Infectious and Tropical Diseases, London School of Hygiene & Tropical Medicine, London, United Kingdom; 3 Department of Molecular Biology & Biotechnology, The Florey Institute, University of Sheffield, Sheffield, United Kingdom; 4 Department of Infection, Immunity and Cardiovascular Diseases, The Florey Institute, University of Sheffield, Sheffield, United Kingdom; Hitit University, Faculty of Medicine, TURKEY

## Abstract

**Background:**

Scabies is a WHO neglected tropical disease common in children in low- and middle-income countries. Excoriation of scabies lesions can lead to secondary pyoderma infection, most commonly by *Staphyloccocus aureus* and *Streptococcus pyogenes* (group A streptococcus, GAS), with the latter linked to acute post-streptococcal glomerulonephritis (APSGN) and potentially rheumatic heart disease (RHD). There is a paucity of data on the prevalence of these skin infections and their bacterial aetiology from Africa.

**Methodology/Principal findings:**

A cross-sectional study, conducted over a four-month period that included the dry and rainy season, was conducted to determine the prevalence of common skin infections in Sukuta, a peri-urban settlement in western Gambia, in children <5 years. Swabs from pyoderma lesions were cultured for *S*. *aureus* and GAS. Of 1441 children examined, 15.9% had scabies (95% CI 12.2–20.4), 17.4% had pyoderma (95% CI 10.4–27.7) and 9.7% had fungal infections (95% CI 6.6–14.0). Scabies was significantly associated with pyoderma (aOR 2.74, 95% CI 1.61–4.67). Of 250 pyoderma swabs, 80.8% were culture-positive for *S*. *aureus*, and 50.8% for GAS. Participants examined after the first rains were significantly more likely to have pyoderma than those examined before (aRR 2.42, 95% CI 1.38–4.23), whereas no difference in scabies prevalence was seen (aRR 1.08, 95% CI 0.70–1.67). Swab positivity was not affected by the season.

**Conclusions/Significance:**

High prevalence of scabies and pyoderma were observed. Pyoderma increased significantly during the rainy season. Given the high prevalence of GAS pyoderma among children, further research on the association with RHD in West Africa is warranted.

## Author summary

Scabies is a WHO neglected tropical disease which is common in low- and middle-income countries with tropical climates, which can lead to serious compilations via secondary bacterial infection of skin lesions. Group A streptococcal skin infection is known to cause kidney disease, but may also contribute to rheumatic heart disease. Despite this, little is known about the prevalence of common paediatric skin infections in sub-Saharan Africa, or which bacteria most commonly cause skin infections. We conducted a cross-sectional study of skin infections in The Gambia into the prevalence of scabies, pyoderma and fungal infections in children <5 years. We examined 1441 children and found a scabies, pyoderma and fungal infection prevalence of 15.9%, 17.4% and 9.7% respectively, with significantly increased presence of bacterial skin infections in the rainy season. Over half of bacterial skin infections were found to be positive for group A streptococcus. Our study shows that scabies and pyoderma occur commonly in young children in The Gambia, representing a significant exposure to group A streptococcus at a young age, and that there is a strong seasonal effect on pyoderma, which may have implications for treatment strategies.

## Introduction

*Streptococcus pyogenes* (group A streptococcus, GAS) is a pathogen that causes a wide spectrum of disease, from superficial skin infections through to invasive sepsis and streptococcal toxic shock syndrome [1]. It continues to cause a significant burden of morbidity and mortality globally, particularly in low- and middle-income countries (LMIC) [2]. It is also associated with autoimmune-mediated, post-infective sequelae including acute post-streptococcal glomerulonephritis (APSGN) and acute rheumatic fever (ARF) leading to chronic rheumatic heart disease (RHD) [3]. RHD has largely disappeared from high-income countries except in neglected groups such as Aboriginal Australians [3, 4], but remains a significant problem in LMIC including those in sub-Saharan Africa (sSA) [5]. The classical understanding of ARF involves an acute GAS pharyngitis infection with specific “rheumatogenic” GAS *emm* serotypes [6, 7]. It is, however, increasingly thought that this explanation is incomplete, with a diversity of *emm* types and GAS skin infections potentially playing an important role in LMIC [8, 9]. This is supported by data from countries where GAS pyoderma and RHD are both highly prevalent, but pharyngitis and GAS pharyngeal carriage are low [3, 10, 11]. A significant proportion of GAS skin infections are attributable to secondary bacterial infection of scabies lesions, a WHO neglected tropical disease, known to be particularly prevalent in young children living in poverty-related conditions in tropical countries [12–14]. Despite this, there are few data sources on scabies epidemiology for sSA [15–20].

Pyoderma (defined as any infection of the skin involving pus) is common in LMIC independently of its association with scabies [12], with over 162 million children estimated to be suffering from impetigo globally [21]. Epidemiological and microbiological data on pyoderma in Africa is scarce [3, 22–24]. Due to the importance of GAS as a pathogen in sSA and M-protein type-specific vaccines in development, a registry of GAS infections in Africa has been initiated to address the paucity of microbiological data [25]. Superficial fungal infections are also common globally, particularly in children in LMIC. The burden in Africa is heterogeneous, with prevalence in schoolchildren ranging from 10–80% [26].

In The Gambia, the prevalence of these common skin infections and the bacterial aetiology of pyoderma in children are unknown. To provide preliminary data, we performed a cross-sectional study to determine prevalence of scabies, pyoderma and fungal skin infections in children aged <5 years in a peri-urban community in western Gambia. We also aimed to characterise the microbiological aetiology of pyoderma in this setting.

## Methods

### Setting

The Gambia is the smallest country by area in mainland Africa, with a population of 1.9 million [27]. It was ranked 174^th^ in the United Nations Human Development Index in 2017 [28]. The annual seasonal climate consists of a long dry season from November to May and a short rainy season between June and October. Sukuta is an area within the West Coast Region peri-urban conurbation, with a population of 47,048 in 2013, including 7,234 children aged under 5 years, and an average household size of 8.1 [27].

### Study design and sampling

A cross-sectional, population-based study was conducted in Sukuta, in children aged <5 years, to determine the prevalence of scabies, pyoderma and fungal infections. Prior to the study, geospatial census data from 2013 was used to divide Sukuta into 37 geographical clusters of approximately 100 households (S1 Fig). In order to minimise selection bias, a one-stage, random cluster sampling method was used, sampling clusters in a randomly generated order. The latest census data (2013) on households was out of date, so was not used as a sampling list in each geographical cluster. Instead, all households in each selected cluster were approached for participation. Eligible participants were children <5 years of age sleeping in a compound within the geographical area of the cluster. Children <5 years were chosen as this age-group are below school age, and therefore more likely to be present during home visits. Participants were examined over a four-month period between May and September 2018. Sample size was determined by availability of study resources.

Information on socio-demographic factors, and possible risk factors for presence of skin infections were collected from participant’s parents prior to examination of the participant, and from the participant’s infant welfare card (if available). Socio-demographics recorded included age, sex, tribal group, mother’s educational level and household size. Possible risk factors and confounders recorded were breastfeeding status, birthweight (if known), household water source and distance, frequency of full body washing and ironing of clothes, whether clean clothes were worn every day, the presence in the compound of a handwashing area, an open fire for cooking, previous history of skin infections, burns, malnutrition or nutritional supplementation.

### Diagnosis, training and case definitions

Study nurses underwent a 2-day training led by a physician, to introduce basic dermatology skills, recognise common paediatric skin complaints, and instruct on use of an adapted IMCI algorithm (S2 Fig) [29]. Participants were examined in the presence of their parents within the participant’s compound. This was often on private verandas, to ensure adequate natural light for skin examination, whilst maintaining privacy as far as possible. Clothes were removed where appropriate, though underwear was not removed unless necessary to closely examine or swab lesions. When examining sensitive areas or at the parents’ or participant’s request, examinations were performed indoors for more privacy. Scabies cases were diagnosed clinically based on the presence of pruritus and papules in a typical distribution. Pyoderma was defined as any skin lesion with evidence of pus or crusts. Infected scabies was diagnosed when scabies was present with evidence of inflammation and pyoderma in the same distribution. Fungal (dermatophyte) skin infection was diagnosed on the basis of the presence of round or oval flat scaly patches, with features typical of tinea infection. Where pyoderma was diagnosed, a wound swab was taken. All skin conditions identified were managed appropriately according to the algorithm and treatment guidelines (S2 Fig and S1 Table). Antibiotics were administered by study nurses in the field and follow-up of all cases was undertaken by the study clinician within one week.

### Algorithm validity

To determine the sensitivity and specificity of the algorithm used, a subset of participants were selected opportunistically and re-examined by a general physician with experience of diagnosing paediatric dermatological conditions in The Gambia, blinded to the nurse’s diagnosis. A sample size of 123 was calculated to be sufficient to detect a sensitivity of 80% compared to the gold standard examination with a precision of 10% assuming a prevalence of 50% of each condition.

### Swab collection and bacteriological methods

Wound swabs were taken to determine the presence of *S. aureus* and/or GAS by microbiological culture. A single swab was taken from the largest present pyoderma lesion; wounds were superficially cleaned with saline and crusts were lifted to swab the base. Samples were collected using Nylon flocked swabs (Copan) stored in liquid Amies transport medium. These were chosen over standard cotton swabs as future molecular/microbiome studies are planned. Swabs were transported in a cold box to the MRC Unit The Gambia at LSHTM (MRCG) for same-day culturing on 5% sheep’s blood agar and incubated at 37°C overnight. Purity plates were done for mixed infection. Identification by catalase and confirmation for either *S*. *aureus* or GAS was done using the Remel Staphaurex Plus and Streptex latex agglutination tests, respectively. Antibiotic sensitivity pattern was obtained using disc diffusion according to CLSI methods [30] in line with the MRCG clinical diagnostic laboratory wound culture standard operating procedures. Only *S*. *aureus* and beta-haemolytic streptococci were considered relevant organisms to record.

### Investigation of seasonality

Data collection spanned both the dry and rainy season (defined as after the 26^th^ of June 2018 when the first rains of the year occurred), allowing for comparison between the prevalence, and the proportion of pyoderma caused by *S*. *aureus* and GAS between these two periods. In addition, the first cluster sampled was subsequently resampled at the end of the study in order to directly compare prevalence in the same population before and after the start of the rainy season.

To further investigate seasonal change in pyoderma in The Gambia, we also leveraged the presence of existing medical records from a rural primary healthcare clinic run by MRCG to perform a post-hoc analysis. The MRC Keneba clinic, in West Kiang, provides free primary care and has been collecting electronic medical records since 2009 [31]. Records on the number of presentations per month of <5 year olds for skin complaints (including impetigo, cutaneous abscess, furuncle or carbuncle, and cellulitis) between 2011 and 2018 were interrogated to ascertain if seasonal variation was observed.

### Data collection and statistical analysis

A questionnaire was delivered to participant’s parents to collect information on socio-demographics and risk factors for skin diseases. Data were collected on tablet computers using REDCap electronic data capture tools hosted at MRCG [32]. Data were analysed using Stata version 15.1. The cluster random sampling method was corrected for using the *svyset* command. Adjusted binomial exact confidence intervals were calculated for prevalence estimates. Associations between socio-demographic and other risk factors and skin infections were investigated using multivariable logistic regression models. Variables and category levels were selected for inclusion using forwards and backwards stepwise regression, without *svyset* correction, using a likelihood ratio test significance level of p<0.2. The multivariable models were then rerun with *svyset* correction to obtain adjusted odds ratios (aOR). All socio-demographics and risk factors were categorical variables, except household size, which was continuous. The population attributable risk (PAR) [33] for scabies as a cause of pyoderma was calculated. The adjusted prevalence ratio (aPR) for the prevalence of skin conditions before and after the start of the rainy season were estimated using a multivariable Poisson regression model, adjusting for socio-demographics, except when comparing the first cluster before and after the rains, which was unadjusted. Predicted probabilities of the presence of skin conditions by week sampled were calculated using the *margins* command following Poisson regression models adjusting for socio-demographics. Sensitivity, specificity and kappa statistic were calculated to determine the accuracy of the diagnostic algorithm compared to the diagnosis reached by the physician. Significance was set at p<0.05.

### Ethical considerations

Ethical approval for the study was provided by The Gambia Government/MRC Joint Ethics Committee (SCC1587). Written or thumb-printed informed consent was obtained in a local language from a parent for all participants included in the prevalence study prior to involvement.

## Results

### Prevalence of skin infections

A total of 1441 participants from 9 clusters were examined between May and September 2018. The mean number of participants per cluster was 160.1 (range 62–306). Overall, 47.9% of participants were male and the mean age was 28.9 (SD 16.7) months. Scabies prevalence was 15.9% (95% CI 12.2–20.4), pyoderma prevalence was 17.4% (95% CI 10.4–27.7), and fungal infection prevalence was 9.7% (95% CI 6.6–14.0). The prevalence of scabies, pyoderma and fungal infections by socio-demographic characteristics is presented in Table 1. Prevalence of co-infections are presented in S2 Table.

**Table 1:** Prevalence of skin infections by socio-demographics.

Socio-demographics		Total	Scabies			Pyoderma			Fungal		
		n	n	%	95% CI	n	%	95% CI	n	%	95% CI
Total		1441	229	15.9	12.2–20.4	251*	17.4	10.4–27.7	140	9.7	6.6–14.0
Sex	Male	690	125	18.1	14.1–23.0	110	15.9	9.8–24.9	89	12.9	9.1–18.0
	Female	751	104	13.8	10.5–18.1	141	18.8	10.6–31.1	51	6.8	4.4–10.3
Age	<1 yr	288	53	18.4	14.4–23.3	21	7.3	3.9–13.3	22	7.6	4.6–12.3
	1–2 yrs	310	57	18.4	13.7–24.3	47	15.2	8.2–26.4	17	5.5	2.8–10.6
	2–3 yrs	320	45	14.1	9.4–20.6	65	20.3	12.7–30.8	32	10.0	5.4–17.8
	3–4 yrs	523	74	14.2	9.8–20.0	118	22.6	12.2–38.0	69	13.2	9.7–17.8
Tribe^	Mandinka	905	135	14.9	11.2–19.6	149	16.5	9.5–27.1	98	10.8	7.1–16.1
	Wolof	151	26	17.2	12.4–23.4	25	16.6	8.0–31.1	13	8.6	4.3–16.4
	Fula	191	36	18.9	12.7–27.0	36	18.9	8.8–35.8	16	8.4	5.5–12.5
	Jola	70	12	17.1	10.0–27.9	12	17.1	9.2–29.8	5	7.1	2.8–16.9
	Serehule	72	16	22.2	14.5–32.6	22	30.6	18.9–45.4	6	8.3	4.1–16.3
	Other^	51	4	7.8	3.8–15.6	7	13.7	6.3–27.2	2	3.9	1.1–13.2
Median household size (IQR)		6 (4–9)	6 (4–9)	-	-	7 (5–9)	-	-	6.5 (5–9)	-	-
Mother’s education†^	None	549	92	16.8	11.3–24.1	112	20.4	11.7–33.1	61	11.7	6.8–19.2
	Arabic school only	99	12	12.1	8.0–17.9	19	19.2	8.7–37.1	6	6.1	3.0–12.0
	Primary only	199	29	14.6	8.9–22.9	31	15.6	7.7–29.0	22	11.1	6.7–17.7
	Secondary only	558	88	15.8	12.9–19.1	82	14.7	9.5–22.1	45	8.1	6.3–10.3
	Higher education	35	8	22.9	10.8–42.1	7	20.0	6.7–46.5	3	8.6	2.3–27.2

Confidence intervals are corrected for cluster sampling design.

*One participant with diagnosed with pyoderma was not swabbed

^One participant with missing data

†English school attendance

### Associations with socio-demographic and other risk factors

Table 2 shows the aOR for skin infection presence by socio-demographic and other risk factors. The odds of scabies and fungal infections were significantly lower in females than males (aOR 0.70, 95% CI 0.61–0.82 and aOR 0.44, 95% CI 0.32–0.61 respectively). Pyoderma increased with age (aOR 3.13 of pyoderma in 3–4 years compared to <1 year, 95% CI 2.00–4.88), was higher in Serehule children compared to Mandinka children (aOR 1.99, 95% CI 1.16–3.41) and increased with household size (aOR 1.03, 95% CI 1.01–1.06). Various behavioural risk factors were identified, including lower odds of scabies in those whose clothes are always ironed (aOR 0.26, 95% CI 0.07–0.98), and higher odds of fungal infections in those not wearing freshly washed clothes every day (aOR 13.5, 95% CI 3.22–26.60). Current breastfeeding was protective against fungal infections, but increased the odds of scabies (aOR 1.67, 95% CI 1.12–2.49). A history of previous skin infections increased the odds of all three infections. Results from the univariable regression models are presented in S3 Table. Results from the forwards and backwards stepwise regression procedures are presented in S5 and S6 Tables.

**Table 2:** Adjusted odds ratios for socio-demographic and other risk factors potentially associated with skin infections.

		Scabies			Pyoderma			Fungal		
		aOR	p value	95% CIs	aOR	p value	95% CIs	aOR	p value	95% CIs
Sex	Male	ref			ref			ref		
	Female	0.70	0.001*	0.61–0.82	1.22	0.201	0.88–1.70	0.44	<0.001**	0.32–0.61
Age category	<1 year	ref			ref			ref		
	1–2 years	0.91	0.634	0.60–1.40	1.98	0.086	0.89–4.43	0.48	0.006*	0.30–0.76
	2–3 years	0.81	0.529	0.38–1.72	2.85	<0.001**	1.90–4.26	0.55	0.026*	0.33–0.91
	3–4 years	0.87	0.670	0.42–1.79	3.13	<0.001**	2.00–4.88	0.79	0.509	0.36–1.74
Tribe	Mandinka	ref			ref			ref		
	Serehule	1.72	0.044*	1.02–2.90	1.99	0.018*	1.16–3.41			
	Other	0.42	0.020*	0.21–0.84				0.39	0.105	0.12–1.28
Mean household size					1.03	0.014*	1.01–1.06			
Mother’s education†	None	ref						ref		
	Arabic school only	0.60	0.007*	0.43–0.83				0.48	0.040*	0.24–0.96
	Secondary only							0.69	0.126	0.42–1.14
Currently breastfeeding	No	ref						ref		
	Yes	1.67	0.017*	1.12–2.49				0.52	0.043*	0.27–0.98
Low birth weight (<2.5kg)	No							ref		
	Yes							1.80	0.006*	1.25–2.59
Water source	Tap				ref					
	Well				1.41	0.434	0.54–3.73			
Clean clothes	Every day							ref		
	Not every day							13.50	0.003*	3.22–56.60
Clothes ironed	Never	ref								
	Always	0.26	0.048*	0.07–0.98						
Handwashing area in compound	No	ref								
	Yes	0.74	0.107	0.50–1.09						
Open fire in compound	No	ref			ref					
	Yes	1.51	0.021*	1.09–2.10	1.40	0.018*	1.08–1.82			
Previous skin infection	None	ref			ref			ref		
	One	3.09	<0.001**	2.19–4.64	1.94	0.002*	1.40–2.69	2.34	0.005*	1.41–3.87
	More than one	4.51	<0.001**	2.63–7.73	2.34	0.002*	1.53–3.57	2.14	0.072	0.92–5.02
History of burn	No	ref								
	Yes	0.65	0.154	0.35–1.22						
History of malnutrition	No				ref					
	Yes				0.67	0.243	0.32–1.40			
History of nutritional supplementation	No	ref								
	Yes	0.32	0.170	0.06–1.83						
									

Reported values are from multivariable logistic regression models, including only variables and category levels selected by stepwise regression with an elimination level of p>0.2. Grey cells represent variables not included in each multivariable analysis. For all three skin infections, forwards and backwards stepwise regression produced the same final model (S5 and S6 Tables). Sex and age group were included in all models, and correction for cluster sampling design was done on the final models. Data from univariable logistic regression models are shown in S3 Table. ref = reference category used; aOR = adjusted odds ratio

*significant at p<0.05

**significant at p<0.001

†English school attendance

The presence of pyoderma was significantly associated with scabies infestation (aOR 2.74, 95% CI 1.61–4.67) as shown in Table 3. The PAR of scabies as a cause of pyoderma was 14.7% (95% CI 5.1–23.3).

**Table 3:** Adjusted odds ratios and population attributable risk percentage for scabies as a cause of pyoderma, *S*. *aureus* positive pyoderma and GAS positive pyoderma.

		Scabies n (%)		aOR (p value, 95% CI)	PAR % (95% CI)
		No	Yes		
All pyoderma	No	1031 (85.1)	159 (69.4)	ref	
	Yes	181 (14.9)	70 (30.6)	2.74* (0.002, 1.61–4.67)	14.7 (5.1–23.3)
*S*. *aureus* positive pyoderma	No	34 (18.9)	14 (20.0)	ref	
	Yes	146 (81.1)	56 (80.0)	1.08 (0.775, 0.61–1.91)	0.4 (-2.7–3.4)
GAS positive pyoderma	No	88 (48.9)	35 (50.0)	ref	
	Yes	92 (51.1)	35 (50.0)	1.26 (0.179, 0.88–1.81)	2.8 (-1.7–7.1)

Reported values are from a multivariable logistic regression model adjusting for sex, age group, tribe, household size and mother’s education, following correction for cluster sampling design. ref = reference category used; aOR = adjusted odds ratio

*significant at p<0.05; PAR = population attributable risk.

### Pyoderma swab culture results

Pyoderma swabs were taken from 250 participants (one participant diagnosed with pyoderma was not swabbed). Overall, either *S*. *aureus* or GAS was cultured from 90.0% of swabs, with 80.8% positive for *S*. *aureus*, and 50.8% for GAS (41.6% were positive for both). Additionally, two samples were positive for other beta-haemolytic streptococci (one group D streptococcus and one non-groupable *Streptococcus* species). The proportion of GAS and *S*. *aureus* causing pyoderma were similar regardless of scabies infection (Table 3). All GAS isolates were sensitive to penicillin, while 99% of *S*. *aureus* isolates were methicillin-sensitive. Antibiotic sensitivities of isolates are presented in S3 and S4 Figs. Swabs taken from the lower limbs were significantly more likely to be positive for GAS than swabs from other sites (S7 Table).

### Seasonal variation of skin infection prevalence

**Fig 1:**
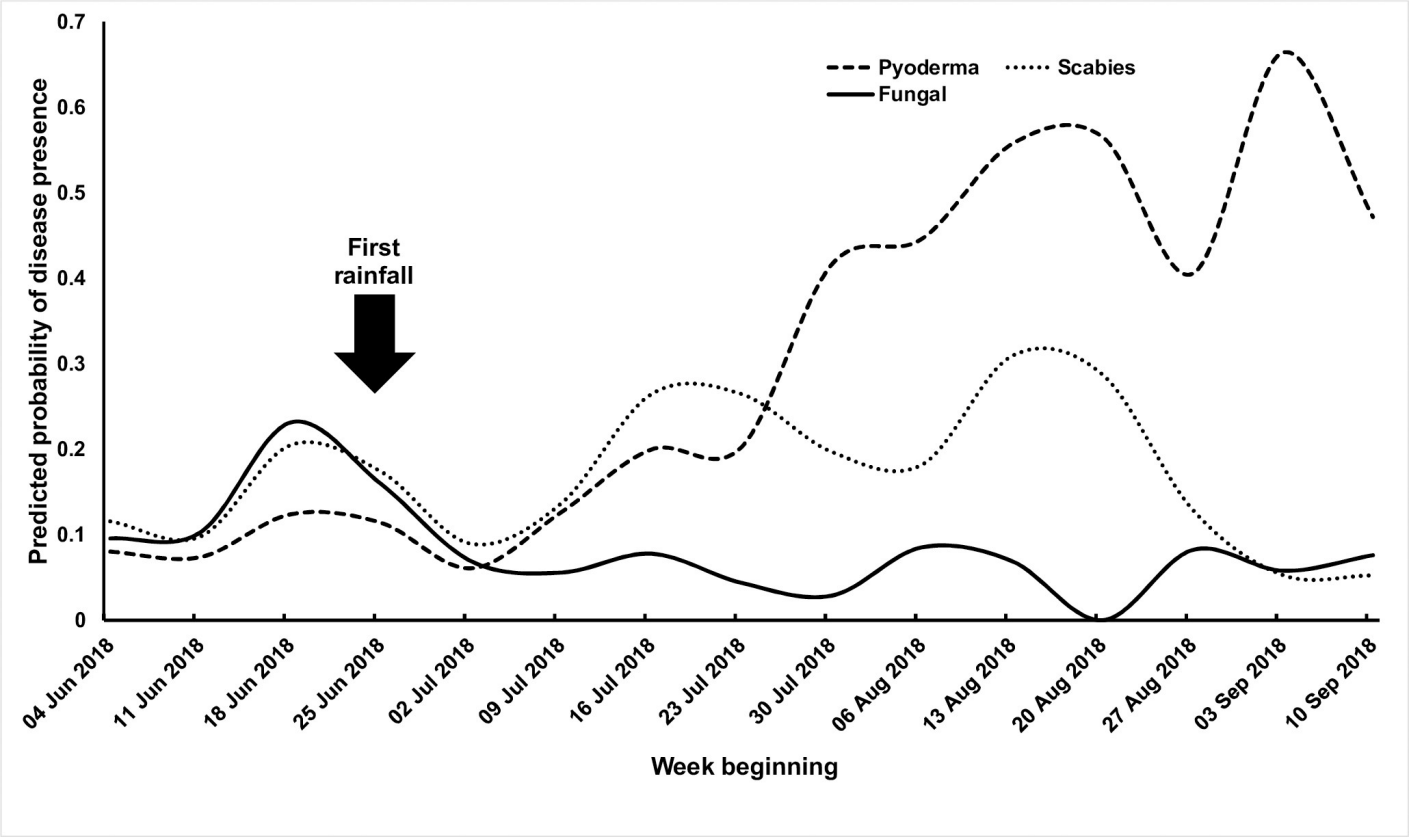
Predicted probabilities of skin infection prevalence by week examined.

The respective prevalence of scabies, pyoderma and fungal infection were 15.3%, 8.9% and 14.4% before the start of the rainy season, compared to 16.3%, 23.1% and 6.6% after. The prevalence of scabies did not change significantly (aPR 1.08, 95% CI 0.70–1.67), whereas pyoderma prevalence significantly increased following the start of the rains (aPR 2.42, 95% CI 1.39–4.23). Fungal infection prevalence fell significantly (aPR 0.44, 95% CI 0.32–0.60) (S8 Table). The PAR of scabies as a cause of pyoderma was 18.5% (95% CI 4.1–30.8) before the start of the rainy season compared to 13.6% (95% CI -0.7–25.8) afterwards. The prevalence of scabies, pyoderma and fungal skin infections by week sampled are presented in Fig 1. The increase in pyoderma prevalence during the rainy season was confirmed by resampling of the first cluster sampled (7.9% vs. 21.7%, PR 2.74, 95% CI 1.23–6.12). The start of the rains did not significantly affect the proportion of *S*. *aureus* or GAS detected (S8 Table).

**Fig 2:**
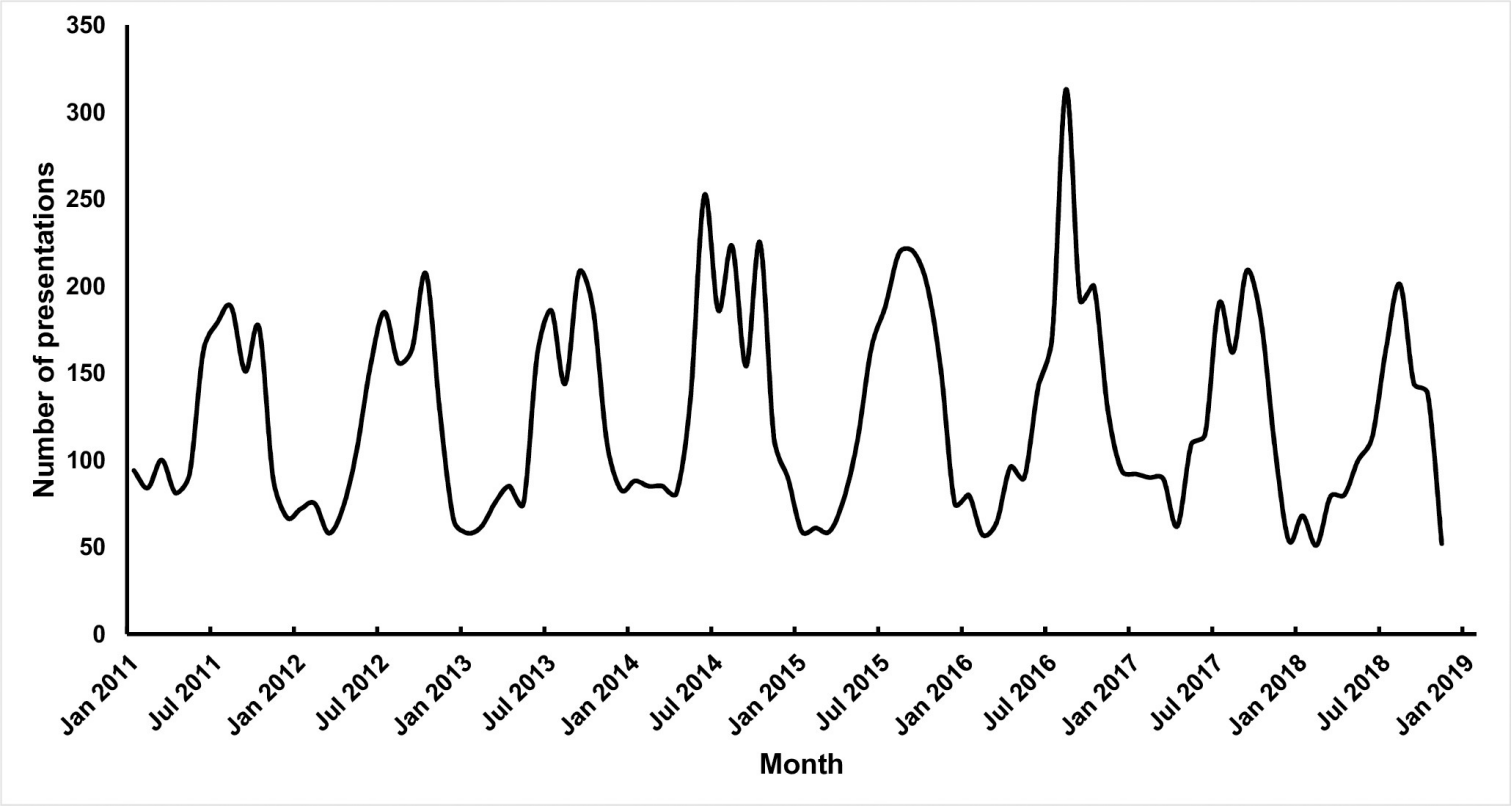
Number of skin complaint presentations in under 5s at MRCG Keneba clinic by month 2011–2018.

The data obtained from the MRCG Keneba clinic records of presentations per month of children <5 for all skin complaints are presented in Fig 2. The number of presentations peaked each year between June and December, coinciding with the rainy season.

### Sensitivity and specificity of the diagnostic algorithm

Diagnosis by nursing staff using the algorithm (S2 Fig) was 97.1% sensitive and 96.6% specific for the diagnosis of pyoderma, and 83.3% sensitive and 97.0% specific for non-infected scabies. Sensitivity and specificity for the pyoderma and scabies co-infection were 81.3% and 97.2% respectively. Sensitivity of detection of fungal infections was lower at 66.7%, but specificity was 95.8%. Full results including the kappa statistic for inter-rater agreement are presented in S9 Table.

## Discussion

We found a high burden of skin infections among Gambian children and that pyoderma increased significantly during the rainy season. The scabies prevalence we observed of 15.9% is consistent with other studies from sSA [15, 16, 18–20], but the overall prevalence of pyoderma of 17.4% is higher than the estimated median impetigo prevalence of 7% for Africa [21]. Studies in other settings have found a strong association between scabies and pyoderma, with one study in Fiji finding a PAR of scabies as a cause of impetigo of 93.1% [10]. We observed a PAR of 14.7%, indicating that scabies may play a less significant role in pyoderma rates in this setting. Interventions to reduce scabies, shown to be effective elsewhere [34–36], may still be worthwhile given the relatively high scabies prevalence.

The 8.9% pyoderma prevalence in children examined before the start of the rainy season was consistent with previous estimates for Africa [21]. However, in those examined during the rainy season, the prevalence rate was significantly higher (23.1%). This effect was confirmed in the cluster that was sampled both before and after the start of the rainy season. Furthermore, a strong seasonal trend was seen at the rural MRCG Keneba clinic in paediatric skin presentations between 2011 and 2018, which supports the hypothesis that bacterial skin infections are seasonal in The Gambia, a finding also observed in 1980 [17].

Very little microbiology data exist from pyoderma cases in Africa [3, 21]. By taking swabs from pyoderma lesions, we were able to provide robust estimates for the prevalence of *S*. *aureus* and GAS pyoderma. 50.8% of swabs were positive for GAS, which is lower than the global median of 74% noted previously from the limited heterogeneous studies available [21]. However, with the high pyoderma prevalence during the rainy season, this still indicates a significant exposure of children <5 to GAS and *S*. *aureus* annually. Better understanding the carriage and transmission of these organisms within households and communities in The Gambia is required to design preventative interventions to reduce the disease burden. Our antimicrobial susceptibility data showed reassuringly low rates of methicillin-resistant *S*. *aureus* which is consistent with other data we and others have recently published [37, 38]. Of greater concern was the resistance to erythromycin (13.3%) and co-trimoxazole (24.2%), especially as MDA of azithromycin is recommended by the WHO to eradicate trachoma and yaws [39, 40], and use may increase in African settings as evidence of impact on <5 mortality increases [41, 42]. As co-trimoxazole is widely used in The Gambia, the reduced susceptibility observed was unsurprising [43].

The reason for the seasonal increase in skin infections is not clear, but may reflect behavioural changes between seasons or environmental changes enhancing bacterial proliferation on skin. Indoor space can be limited within households, and during the rainy season people may spend more time in overcrowded indoor areas, increasing skin-to-skin contact. This could partly explain the observed increase, although scabies prevalence did not increase correspondingly as would be expected. Data were not collected on insect bites, but increased insect activity during the rainy season may play a more significant role than scabies in this setting. Further studies are required to explore the exact mechanism for this observation. We also observed associations between increased age, household size, history of previous infections, and certain tribal groups with higher pyoderma rates, some of which have been observed elsewhere [13, 44]. These suggest that individual’s behaviour may also be important in pyoderma transmission.

Additionally, associations between scabies and fungal infections and other risk factors were seen. Current breastfeeding increased the odds of scabies, which may reflect higher risk due to greater skin contact with mothers who may be the source of infection. Ironing of clothes reduced the odds of scabies, which could suggest that some transmission of mites may occur through clothes, in spite of the common understanding that scabies is transmitted only through direct contact. The largest effect-size was seen in participants not wearing freshly washed clothes every day, which increased the odds of fungal infections. These may represent potential targets for behavioural interventions to reduce these skin infections.

In low-resourced healthcare settings, nurses can be expected to diagnose and treat common conditions with little or no dermatology training [21, 29]. Nurses were used in our study for the diagnosis of the skin conditions rather than physicians to determine whether training in the use of a diagnostic algorithm for skin conditions could be effective in this setting. The sensitivity and specificity of the algorithm used were consistent with the validation study [29], confirming that such algorithms for nurses can be effective diagnostic tools. Wider introduction and evaluation of such algorithms could be carried out in The Gambia and similar settings, with a view to increasing diagnosis and treatment, cost-effectiveness, and the effects on other GAS disease. An impact on reducing inappropriate antibiotic prescribing

For pyoderma in the absence of guidelines should also be considered, given the likely contribution to antimicrobial resistance [45].

Our study has several limitations. We sampled from one peri-urban population, so our findings may not be generalisable, particularly to rural settings, where the majority of Gambians live. Furthermore, as we sampled children <5 in this study due to practical constraints, we were unable to include older school-age children, in whom pyoderma prevalence may be higher [10, 22]. Older children would be an important consideration in future studies, especially as children <5 years would currently be excluded from mass drug administration (MDA) of ivermectin for scabies due to weight-based dosing rules [46]. This younger age group may, however, benefit from MDA with antibiotics such as azithromycin to reduce the burden of pyoderma [47, 48].

No up-to-date list of households or residents was available for the area, and it was not feasible to enumerate the area in advance of the study. We therefore used a random cluster sampling method, dividing Sukuta into clusters of approximately 100 households based on 2013 census data, without detailed within household population metrics. This reduced the precision of the prevalence confidence intervals and prevented analysis of results with respect to households. Accordingly, we also did not have accurate data on children missing during study visits, which had the potential to introduce some selection bias. The study was conducted over four months, so we were unable to determine whether the observed seasonal variation is cyclical on an annual basis or changes year-to-year. We were only able to resample one cluster to compare prevalence directly, and given that prevalence varied by cluster, we should interpret the results cautiously. Additionally, the associations observed between risk factors and skin infections should be interpreted with caution as question responses were open to recall bias, and there may have been residual confounding factors not measured. Finally, the subset used for the algorithm validation sub-study were selected opportunistically, which may have been open to selection bias. Despite these limitations, this study provides good baseline data for the prevalence of these common skin infections in a country where no other recent data exists and also provides valuable microbiological data on skin pathogens, which is lacking from LMIC [1–3].

This study has shown that skin infections are common in The Gambia, particularly pyoderma caused by *S*. *aureus* and GAS during the rainy season. Skin infections may be overlooked, particularly in LMIC where there are other pressing health concerns, but with such high prevalence of pyoderma, they may represent a crucial early exposure to potentially serious pathogens. The high incidence of invasive *S*. *aureus* infections in Gambian children is increasingly recognised and can be triggered by skin infections [38]. The exact mechanism by which RHD follow GAS infection is still not understood, but repeated GAS skin infections may be important [49]. It is plausible that exposure to GAS through the skin at a young age may be a step in the aetiology of RHD [8, 9], particularly as GAS pyoderma is known to trigger the better understood, immune-complex mediated APSGN, which is also epidemiologically linked to scabies [23, 50].

### Conclusions

Treating skin infections for their own sake is justification enough, but since GAS and *S*. *aureus* pyoderma can lead to serious disease, diagnosing and treating them as an upstream target to prevent invasive infections and non-suppurative complications may also be a cost-effective way to prevent these more serious conditions. Increased research into GAS carriage, transmission dynamics, and correlates of protection from disease is therefore warranted in African settings.

## Supporting information

S1 Checklist STROBE checklist. (DOCX)

S1 Fig The Sukuta area divided into 37 geographical clusters with approximately equal number of compounds from 2013 geospatial census data, used for one-stage cluster sampling method. (TIF)

S2 Fig Diagnostic algorithm for common skin conditions adapted from IMCI algorithm designed and validated by Steer et al. (2009). (TIF)

S3 Fig *Staphylococcus aureus* isolate antibiotic sensitivities. (TIF)

S4 Fig Group A streptococcus isolate antibiotic sensitivities. (TIF)

S1 Table Treatment guidelines used for common skin conditions. (DOCX)

S2 Table Prevalence of infected scabies, and co-infection with scabies, pyoderma and fungal infections. (DOCX)

S3 Table Odds ratios for socio-demographic and other risk factors potentially associated with skin infections in univariable logistic regression models. (DOCX)

S4 Table Adjusted odds ratios for socio-demographic and other risk factors potentially associated with skin infections in global multivariable logistic regression models including all variables. (DOCX)

S5 Table Forwards stepwise logistic regression steps. (DOCX)

S6 Table Backwards elimination stepwise logistic regression steps. (DOCX)

S7 Table Swab positivity by swab site. (DOCX)

S8 Table Prevalence rate ratios for presence of skin infections by whether examined before or after the start of the rainy season using Poisson regression. (DOCX)

S9 Table Sensitivity, specificity and kappa statistic for each skin diagnosis made by trained nurses using the adapted skin condition diagnostic algorithm as compared with the diagnosis of a physician. (DOCX)

S1 Dataset Main study dataset. (XLSX)

S2 Dataset Skin complaint presentation dataset from MRCG Keneba clinic. (XLSX)
