# Supplementary figures and images for: High burden and seasonal variation of paediatric scabies and pyoderma prevalence in The Gambia: A cross-sectional study
Source: PLoS Negl Trop Dis. 2019 Oct 14;13(10):e0007801. doi: 10.1371/journal.pntd.0007801 (PMC6812840; doi:10.1371/journal.pntd.0007801)

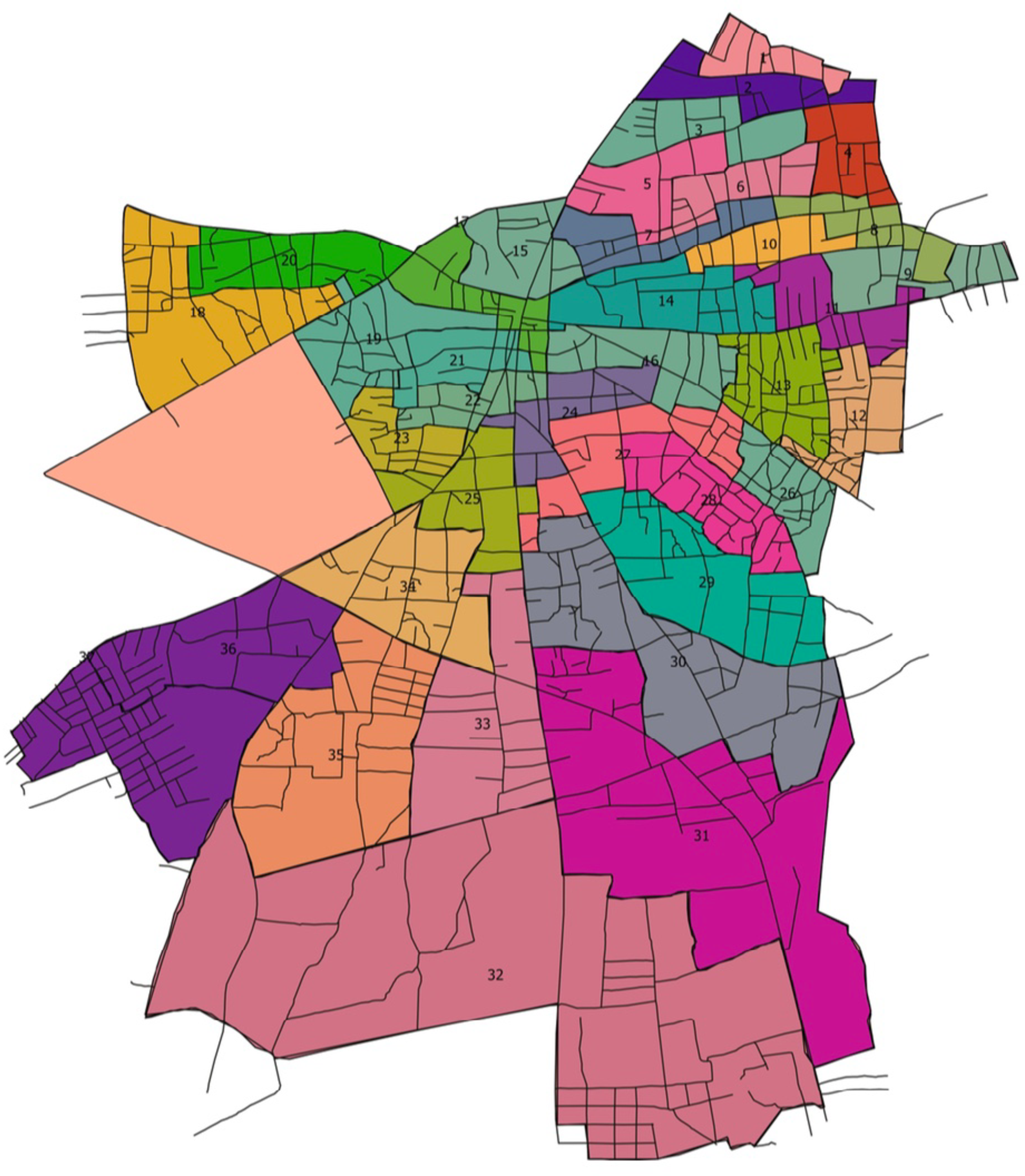

Supplement: S1 Fig — (TIF) [file pntd.0007801.s002.tif]

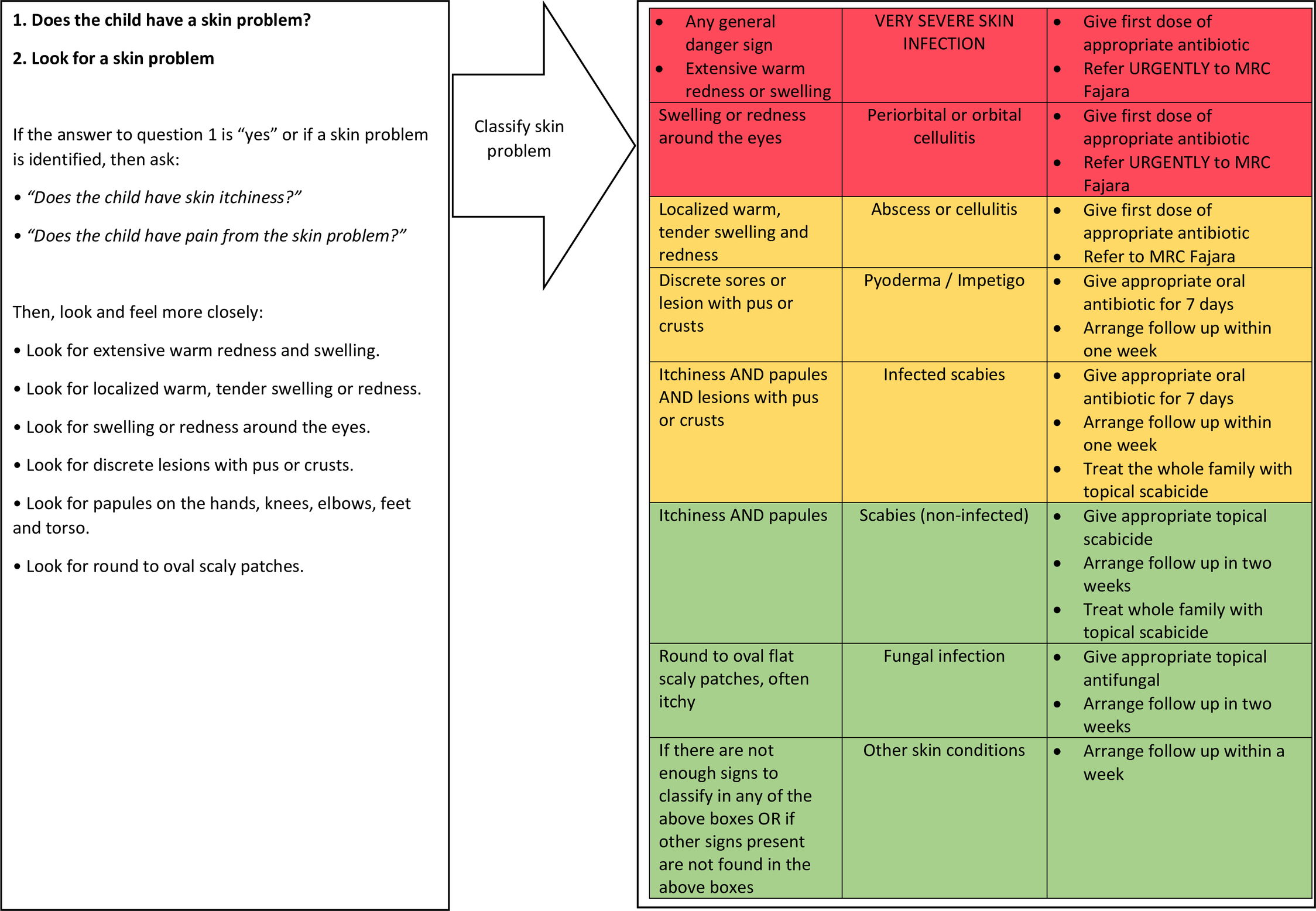

Supplement: S2 Fig — (TIF) [file pntd.0007801.s003.tif]

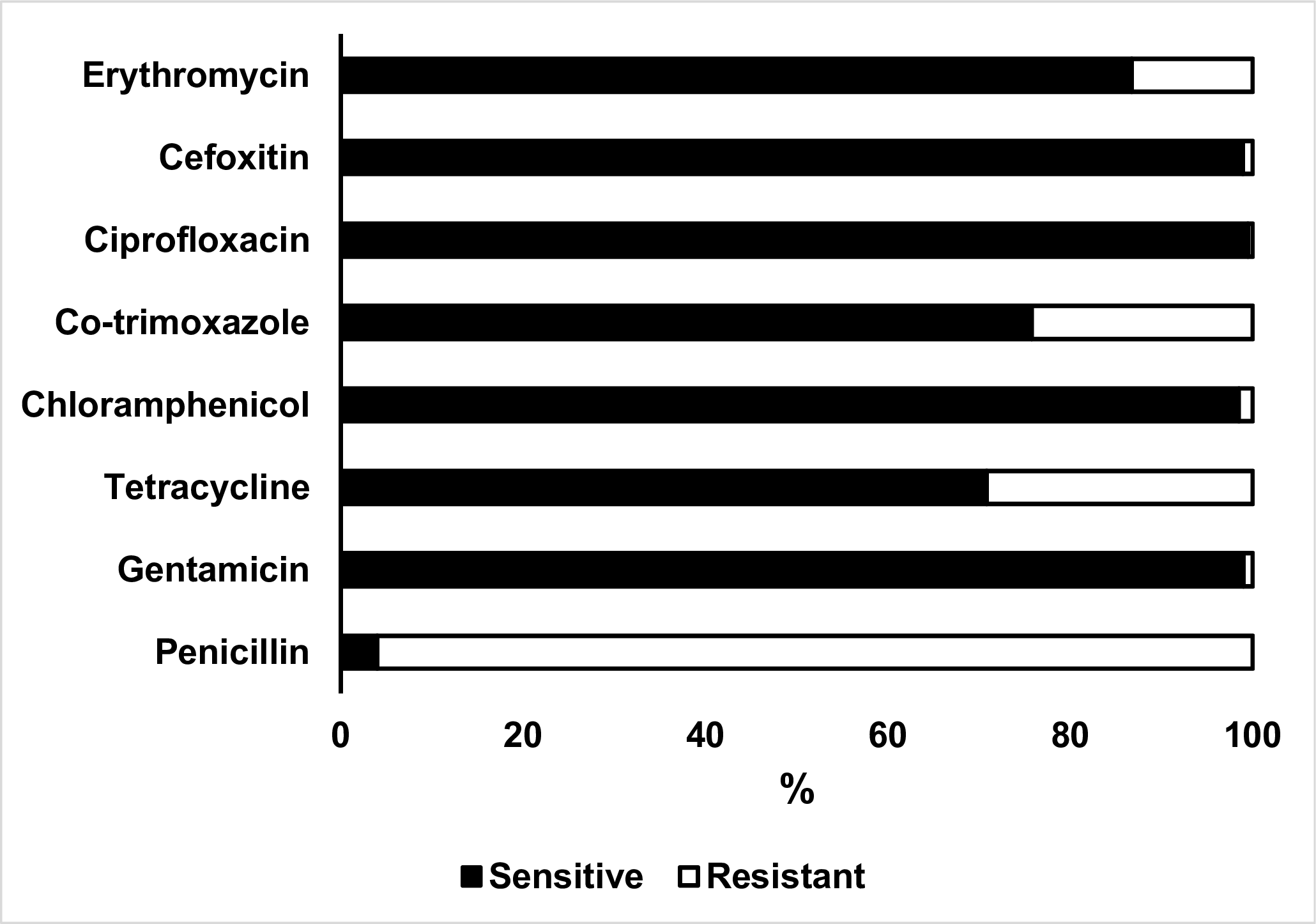

Supplement: S3 Fig — (TIF) [file pntd.0007801.s004.tif]

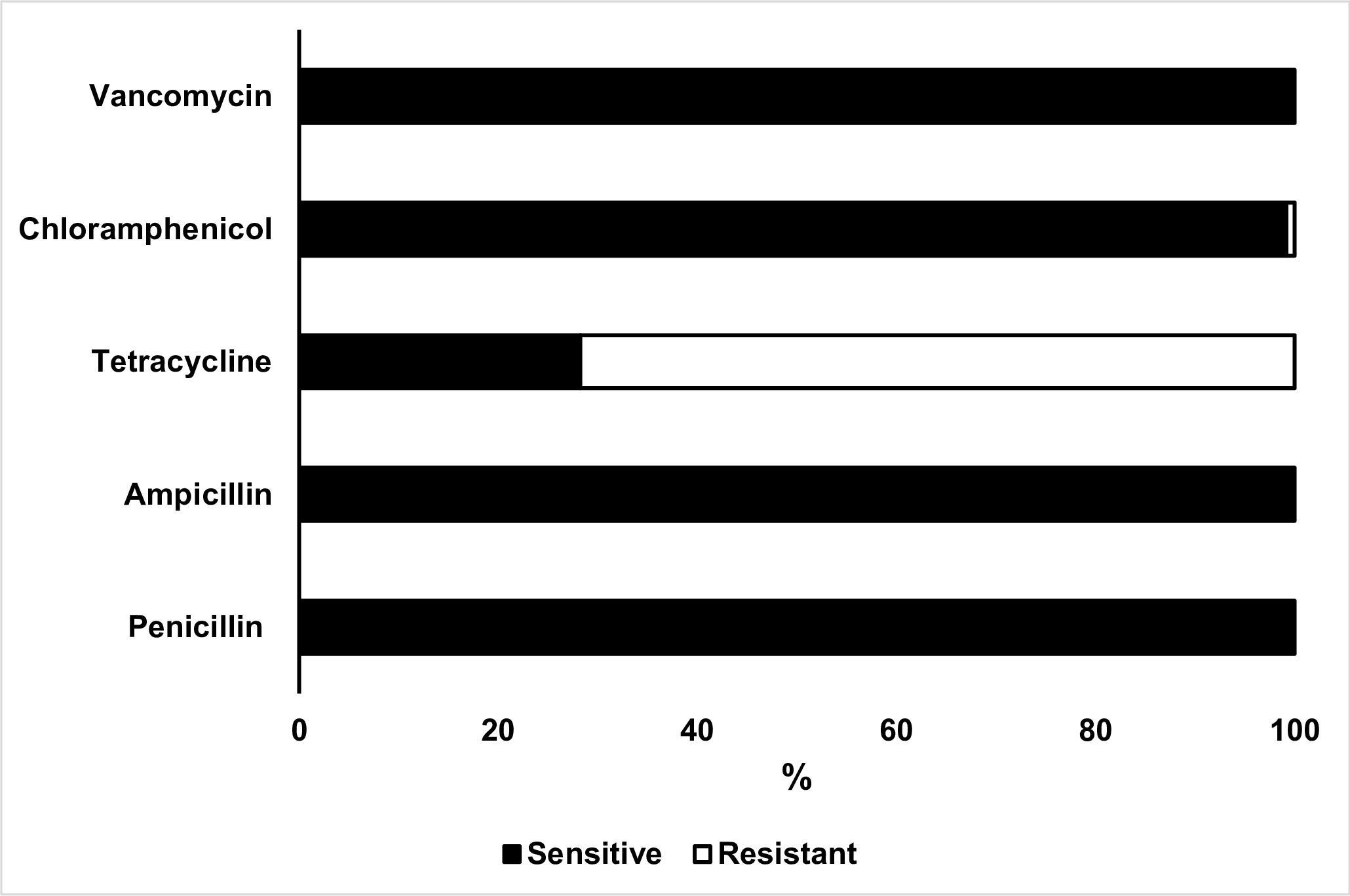

Supplement: S4 Fig — (TIF) [file pntd.0007801.s005.tif]
