# Supplementary material for: High burden and seasonal variation of paediatric scabies and pyoderma prevalence in The Gambia: A cross-sectional study
Source: PLoS Negl Trop Dis. 2019 Oct 14;13(10):e0007801. doi: 10.1371/journal.pntd.0007801 (PMC6812840; doi:10.1371/journal.pntd.0007801)
Supplement: S1 Table — (DOCX) [file pntd.0007801.s006.docx]

| **Diagnosis** | **Drug** | **Age** | **Dosage and course length** |
| --- | --- | --- | --- |
| Very severe skin infection / Periorbital or orbital cellulitis / Abscess or cellulitis | Cloxacillin syrup | Under 28 days | 25mg/kg stat oral dose before referral |
|  |  | 1 month to 2 years | 62.5mg stat oral dose  before referral |
|  |  | 2 - 4 years | 125mg stat oral dose  before referral |
| Alternative (if penicillin allergy) | Azithromycin syrup | All | 12mg/kg once a day for 5 days |
|  |  |  |  |
| Pyoderma / Impetigo / Infected scabies* | Cloxacillin syrup | Under 7 days | 25mg/kg twice a day for one week |
|  |  | 7-20 days | 25mg/kg three times a day for one week |
|  |  | 21-28 days | 25mg/kg four times a day for one week |
|  |  | 1 month to 2 years | 62.5mg four times a day for one week |
|  |  | 2 - 4 years | 125mg four times a day for one week |
| Alternative (if penicillin allergy) | Azithromycin syrup | All | 12mg/kg once a day for 5 days |
|  |  |  |  |
| Scabies (non-infected)** | Benzyl benzoate 25% w/v emulsion | Under 1 year | Dilute with 3 parts water, apply once and wait for 24 hours before washing |
|  |  | 1 - 18 years | Dilute with equal parts water, apply once and wait for 24 hours before washing |
|  |  | Adults (family members) | Do not dilute, apply once and wait for hour hours before washing |
|  |  |  |  |
| Fungal infection | Clotrimazole 1% cream | All | Apply twice a day for two weeks |

*Where participants were diagnosed with infected scabies they were not treated with benzyl benzoate until after the bacterial infection had resolved **Where participant were diagnosed with non-infected or infected scabies all close family contacts were also treated.
