# Supplementary material for: High burden and seasonal variation of paediatric scabies and pyoderma prevalence in The Gambia: A cross-sectional study
Source: PLoS Negl Trop Dis. 2019 Oct 14;13(10):e0007801. doi: 10.1371/journal.pntd.0007801 (PMC6812840; doi:10.1371/journal.pntd.0007801)
Supplement: S2 Table — (DOCX) [file pntd.0007801.s007.docx]

| n=1441 | **n** | **%** | **CI** |
| --- | --- | --- | --- |
|  |  |  |  |
| Any skin infection | 532 | 36.9 | 34.4-39.5 |
| Scabies only | 139 | 9.7 | 8.2-11.3 |
| Pyoderma only | 163 | 11.3 | 9.7-13.1 |
| Fungal only | 99 | 6.9 | 5.6-8.3 |
| Infected scabies* | 25 | 1.7 | 1.1-2.6 |
| Scabies and pyoderma co-infection** | 45 | 3.1 | 2.3-4.2 |
| Scabies and fungal co-infection | 22 | 1.5 | 1.0-2.3 |
| Pyoderma and fungal co-infection | 17 | 1.2 | 0.7-1.9 |
| Scabies, pyoderma and fungal co-infection | 4 | 0.3 | 0.1-0.7 |

*Infected scabies defined as scabies with inflammation and pus present in the same distribution. **Scabies and pyoderma co-infection defined as scabies present with pyoderma in a different, non-overlapping location or distribution.
