# Supplementary material for: High burden and seasonal variation of paediatric scabies and pyoderma prevalence in The Gambia: A cross-sectional study
Source: PLoS Negl Trop Dis. 2019 Oct 14;13(10):e0007801. doi: 10.1371/journal.pntd.0007801 (PMC6812840; doi:10.1371/journal.pntd.0007801)
Supplement: S3 Table — (DOCX) [file pntd.0007801.s008.docx]

|  |  | **Scabies** | | | **Pyoderma** | | | **Fungal** | | |
| --- | --- | --- | --- | --- | --- | --- | --- | --- | --- | --- |
|  |  | **OR** | **p value** | **95% CIs** | **OR** | **p value** | **95% CIs** | **OR** | **p value** | **95% CIs** |
|  |  |  |  |  |  |  |  |  |  |  |
| Sex | Male | ref |  |  | ref |  |  | ref |  |  |
|  | Female | 0.73 | <0.001** | 0.65-0.81 | 1.22 | 0.226 | 0.86-1.73 | 0.49 | <0.001** | 0.39-0.63 |
|  |  |  |  |  |  |  |  |  |  |  |
| Age category | <1 year | ref |  |  | ref |  |  | ref |  |  |
|  | 1-2 years | 1.00 | 0.995 | 0.68-1.46 | 2.27 | 0.043* | 1.03-5.00 | 0.70 | 0.192 | 0.40-1.24 |
|  | 2-3 years | 0.73 | 0.151 | 0.46-1.16 | 3.24 | <0.001** | 2.21-4.75 | 1.34 | 0.144 | 0.88-2.04 |
|  | 3-4 years | 0.73 | 0.169 | 0.45-1.18 | 3.70 | <0.001** | 2.39-5.73 | 1.84 | 0.005* | 1.28-2.64 |
|  |  |  |  |  |  |  |  |  |  |  |
| Tribe | Mandinka | ref |  |  | ref |  |  | ref |  |  |
|  | Wolof | 1.19 | 0.252 | 0.86-1.63 | 1.01 | 0.971 | 0.67-1.52 | 0.78 | 0.348 | 0.43-1.40 |
|  | Fula | 1.32 | 0.277 | 0.76-2.31 | 1.18 | 0.644 | 0.54-2.59 | 0.75 | 0.128 | 0.51-1.11 |
|  | Jola | 1.18 | 0.469 | 0.71-1.95 | 1.05 | 0.901 | 0.44-2.52 | 0.63 | 0.256 | 0.27-1.50 |
|  | Serehule | 1.63 | 0.069 | 0.95-2.79 | 2.23 | 0.015* | 1.22-4.08 | 0.75 | 0.458 | 0.32-1.76 |
|  | Other | 0.49 | 0.084 | 0.21-1.13 | 0.81 | 0.624 | 0.31-2.13 | 0.34 | 0.036 | 0.12-0.91 |
|  |  |  |  |  |  |  |  |  |  |  |
| Mean household size | | 1.02 | 0.209 | 0.99-1.04 | 1.03 | 0.029* | 1.00-1.05 | 1.02 | 0.036* | 1.00-1.04 |
|  |  |  |  |  |  |  |  |  |  |  |
| Mother’s education† | None | ref |  |  | ref |  |  | ref |  |  |
|  | Arabic school only | 0.69 | 0.048* | 0.47-1.00 | 0.93 | 0.829 | 0.42-2.04 | 0.49 | 0.074 | 0.22-1.09 |
|  | Primary only | 0.85 | 0.577 | 0.44-1.63 | 0.72 | 0.280 | 0.37-1.38 | 0.94 | 0.795 | 0.53-1.57 |
|  | Secondary only | 0.93 | 0.675 | 0.63-1.37 | 0.67 | 0.061 | 0.44-1.02 | 0.66 | 0.061 | 0.43-1.03 |
|  | Higher education | 1.47 | 0.197 | 0.78-2.77 | 0.98 | 0.942 | 0.46-2.09 | 0.71 | 0.610 | 0.16-3.14 |
|  |  |  |  |  |  |  |  |  |  |  |
| Currently breastfeeding | No | ref |  |  | ref |  |  | ref |  |  |
|  | Yes | 1.47 | 0.033* | 1.04-2.08 | 0.42 | <0.001** | 0.32-0.54 | 0.49 | 0.003* | 0.33-0.72 |
|  |  |  |  |  |  |  |  |  |  |  |
| Low birth weight (<2.5kg) | No | ref |  |  | ref |  |  | ref |  |  |
|  | Yes | 1.17 | 0.227 | 0.88-1.56 | 1.09 | 0.707 | 0.66-1.79 | 1.33 | 0.128 | 0.90-1.98 |
|  | Unknown | 1.08 | 0.562 | 0.80-1.45 | 1.50 | 0.002* | 1.22-1.85 | 1.19 | 0.309 | 0.83-1.70 |
|  |  |  |  |  |  |  |  |  |  |  |
| Water source | Tap | ref |  |  | ref |  |  | ref |  |  |
|  | Borehole | 1.30 | 0.390 | 0.67-2.52 | 0.99 | 0.988 | 0.28-3.49 | 1.00 | 0.988 | 0.55-1.80 |
|  | Well | 1.36 | 0.350 | 0.67-2.76 | 1.48 | 0.371 | 0.57-3.87 | 1.11 | 0.645 | 0.66-1.87 |
|  |  |  |  |  |  |  |  |  |  |  |
| Water distance | Inside compound | ref |  |  | ref |  |  | ref |  |  |
|  | <5 mins away | 0.51 | 0.122 | 0.20-1.25 | 1.04 | 0.910 | 0.50-2.17 | 1.21 | 0.499 | 0.65-2.23 |
|  | 5-10 mins away | 1.05 | 0.823 | 0.63-1.77 | 1.27 | 0.199 | 0.86-1.89 | 0.86 | 0.039 | 0.74-0.99 |
|  | >10 mins away | 1.20 | 0.714 | 0.40-3.62 | 0.65 | 0.379 | 0.23-1.88 | 0.66 | 0.296 | 0.278-1.56 |
|  |  |  |  |  |  |  |  |  |  |  |
| Full body wash | Every day | ref |  |  | ref |  |  | ref |  |  |
|  | Not every day | 0.75 | 0.686 | 0.16-3.55 | 0.68 | 0.605 | 0.13-3.62 | NA | NA | NA |
|  |  |  |  |  |  |  |  |  |  |  |
| Clean clothes | Every day | ref |  |  | ref |  |  | ref |  |  |
|  | Not every day | 0.48 | 0.374 | 0.08-2.91 | NA | NA | NA | 13.63 | 0.003* | 3.23-57.48 |
|  |  |  |  |  |  |  |  |  |  |  |
| Clothes ironed | Never | ref |  |  | ref |  |  | ref |  |  |
|  | Sometimes | 0.88 | 0.176 | 0.73-1.07 | 1.40 | 0.005* | 1.14-1.83 | 1.59 | 0.039* | 1.03-2.44 |
|  | Always | 0.18 | 0.011* | 0.06-0.60 | NA | NA | NA | 2.00 | 0.180 | 0.67-5.96 |
|  |  |  |  |  |  |  |  |  |  |  |
| Handwashing area in compound | No | ref |  |  | ref |  |  | ref |  |  |
|  | Yes | 0.71 | 0.046* | 0.50-0.99 | 0.78 | 0.106 | 0.57-1.07 | 0.87 | 0.352 | 0.64-1.20 |
|  |  |  |  |  |  |  |  |  |  |  |
| Open fire in compound | No | ref |  |  | ref |  |  | ref |  |  |
|  | Yes | 1.49 | 0.004* | 1.18-1.88 | 1.35 | 0.034* | 1.03-1.77 | 1.34 | 0.347 | 0.68-2.64 |
|  |  |  |  |  |  |  |  |  |  |  |
| Previous skin infection | None | ref |  |  | ref |  |  | ref |  |  |
|  | One | 2.68 | <0.001** | 1.99-3.60 | 2.21 | 0.001* | 1.58-3.10 | 2.32 | 0.002* | 1.49-3.63 |
|  | More than one | 3.33 | 0.001* | 1.99-5.60 | 2.71 | 0.001* | 1.79-4.11 | 2.47 | 0.001* | 1.31-4.66 |
|  |  |  |  |  |  |  |  |  |  |  |
| History of burn | No | ref |  |  | ref |  |  | ref |  |  |
|  | Yes | 0.78 | 0.381 | 0.43-1.44 | 1.32 | 0.062 | 0.98-1.76 | 0.91 | 0.692 | 0.55-1.52 |
|  |  |  |  |  |  |  |  |  |  |  |
| History of malnutrition | No | ref |  |  | ref |  |  | ref |  |  |
|  | Yes | 0.75 | 0.345 | 0.39-1.44 | 0.84 | 0.593 | 0.42-1.70 | 1.77 | 0.066 | 0.95-3.28 |
|  |  |  |  |  |  |  |  |  |  |  |
| History of nutritional supplementation | No | ref |  |  | ref |  |  | ref |  |  |
|  | Yes | 0.48 | 0.250 | 0.12-1.89 | 0.95 | 0.920 | 0.28-3.20 | 1.33 | 0.410 | 0.62-2.86 |

All values were corrected for cluster sampling design. OR = odds ratio; ref = reference category used; NA = regression analysis not possible due to too few participants; *significant at p<0.05; **significant at p<0.001
