# Supplementary material for: High burden and seasonal variation of paediatric scabies and pyoderma prevalence in The Gambia: A cross-sectional study
Source: PLoS Negl Trop Dis. 2019 Oct 14;13(10):e0007801. doi: 10.1371/journal.pntd.0007801 (PMC6812840; doi:10.1371/journal.pntd.0007801)
Supplement: S4 Table — (DOCX) [file pntd.0007801.s009.docx]

|  |  | **Scabies** | | | | **Pyoderma** | | | | **Fungal** | | | |
| --- | --- | --- | --- | --- | --- | --- | --- | --- | --- | --- | --- | --- | --- |
|  |  | **OR** | **p value** | **95% CIs** | **LR test†** | **OR** | **p value** | **95% CIs** | **LR test†** | **OR** | **p value** | **95% CIs** | **LR test†** |
|  |  |  |  |  |  |  |  |  |  |  |  |  |  |
| Sex | Male | ref |  |  |  | ref |  |  |  | ref |  |  |  |
|  | Female | 0.69 | 0.001 | 0.59-0.82 | 0.0174 | 1.21 | 0.188 | 0.89-1.64 | 0.2001 | 0.45 | 0.001 | 0.30-0.65 | <0.0001 |
|  |  |  |  |  |  |  |  |  |  |  |  |  |  |
| Age category | <1 year | ref |  |  |  | ref |  |  |  | ref |  |  |  |
|  | 1-2 years | 0.92 | 0.659 | 0.60-1.40 |  | 1.81 | 0.197 | 0.68-4.81 |  | 0.43 | 0.001 | 0.28-0.65 |  |
|  | 2-3 years | 0.81 | 0.545 | 0.37-1.77 |  | 2.08 | 0.103 | 0.83-5.22 |  | 0.45 | 0.006 | 0.26-0.74 |  |
|  | 3-4 years | 0.90 | 0.766 | 0.40-2.02 | 0.9314 | 2.27 | 0.148 | 0.70-7.36 | 0.1922 | 0.62 | 0.225 | 0.27-1.43 | 0.0681 |
|  |  |  |  |  |  |  |  |  |  |  |  |  |  |
| Tribe | Mandinka | ref |  |  |  | ref |  |  |  | ref |  |  |  |
|  | Wolof | 1.33 | 0.103 | 0.93-1.90 |  | 1.08 | 0.715 | 0.67-1.74 |  | 0.74 | 0.367 | 0.36-1.52 |  |
|  | Fula | 1.39 | 0.168 | 0.84-2.30 |  | 1.20 | 0.580 | 0.57-2.52 |  | 0.73 | 0.071 | 0.51-1.03 |  |
|  | Jola | 1.11 | 0.79 | 0.57-2.18 |  | 1.14 | 0.741 | 0.46-2.83 |  | 0.58 | 0.341 | 0.17-2.01 |  |
|  | Serehule | 1.93 | 0.035 | 1.06-3.50 |  | 1.90 | 0.046 | 1.01-3.55 |  | 0.58 | 0.229 | 0.22-1.52 |  |
|  | Other | 0.47 | 0.036 | 0.23-0.94 | 0.1089 | 0.83 | 0.663 | 0.31-2.19 | 0.3841 | 0.30 | 0.068 | 0.08-1.12 | 0.2776 |
|  |  |  |  |  |  |  |  |  |  |  |  |  |  |
| Mean household size | | 1.02 | 0.202 | 0.99-1.05 | 0.2617 | 1.04 | 0.013 | 1.01-1.07 | 0.0130 | 1.01 | 0.608 | 0.98-1.03 | 0.7919 |
|  |  |  |  |  |  |  |  |  |  |  |  |  |  |
| Mother’s education† | None | ref |  |  |  | ref |  |  |  | ref |  |  |  |
|  | Arabic school only | 0.58 | 0.029 | 0.37-0.93 |  | 1.05 | 0.913 | 0.42-2.59 |  | 0.41 | 0.020 | 0.20-0.84 |  |
|  | Primary only | 0.89 | 0.685 | 0.47-1.68 |  | 0.85 | 0.567 | 0.46-1.58 |  | 0.85 | 0.468 | 0.53-1.38 |  |
|  | Secondary only | 1.08 | 0.693 | 0.70-1.68 |  | 0.84 | 0.375 | 0.54-1.30 |  | 0.65 | 0.094 | 0.38-1.10 |  |
|  | Higher education | 1.51 | 0.321 | 0.61-3.73 | 0.3484 | 1.40 | 0.325 | 0.67-2.95 | 0.6973 | 0.57 | 0.457 | 0.11-3.02 | 0.1443 |
|  |  |  |  |  |  |  |  |  |  |  |  |  |  |
| Currently breastfeeding | No | ref |  |  |  | ref |  |  |  | ref |  |  |  |
|  | Yes | 1.69 | 0.024 | 1.09-2.61 | 0.0855 | 0.68 | 0.341 | 0.28-1.64 | 0.2085 | 0.50 | 0.063 | 0.24-1.05 | 0.1243 |
|  |  |  |  |  |  |  |  |  |  |  |  |  |  |
| Low birth weight (<2.5kg) | No | ref |  |  |  | ref |  |  |  | ref |  |  |  |
|  | Yes | 1.43 | 0.046 | 1.01-2.03 |  | 1.22 | 0.484 | 0.65-2.31 |  | 1.86 | 0.002 | 1.36-2.55 |  |
|  | Unknown | 1.22 | 0.227 | 0.86-1.73 | 0.3203 | 1.18 | 0.113 | 0.95-1.47 | 0.5205 | 1.04 | 0.765 | 0.77-1.41 | 0.2412 |
|  |  |  |  |  |  |  |  |  |  |  |  |  |  |
| Water source | Tap | ref |  |  |  | ref |  |  |  | ref |  |  |  |
|  | Borehole | 1.24 | 0.326 | 0.77-1.98 |  | 1.00 | 0.994 | 0.30-3.33 |  | 1.14 | 0.733 | 0.48-2.71 |  |
|  | Well | 1.21 | 0.462 | 0.67-2.12 | 0.6726 | 1.29 | 0.551 | 0.50-3.33 | 0.5373 | 1.08 | 0.754 | 0.63-1.85 | 0.9491 |
|  |  |  |  |  |  |  |  |  |  |  |  |  |  |
| Water distance | Inside compound | ref |  |  |  | ref |  |  |  | ref |  |  |  |
|  | <5 mins away | 0.42 | 0.075 | 0.16-1.12 |  | 0.88 | 0.706 | 0.40-1.91 |  | 1.21 | 0.565 | 0.58-2.56 |  |
|  | 5-10 mins away | 0.91 | 0.724 | 0.50-1.64 |  | 1.10 | 0.710 | 0.63-1.92 |  | 0.88 | 0.317 | 0.66-1.17 |  |
|  | >10 mins away | 1.22 | 0.687 | 0.41-3.66 | 0.2097 | 0.60 | 0.239 | 0.23-1.52 | 0.5685 | 0.45 | 0.091 | 0.17-1.17 | 0.4767 |
|  |  |  |  |  |  |  |  |  |  |  |  |  |  |
| Full body wash | Every day | ref |  |  |  | ref |  |  |  | ref |  |  |  |
|  | Not every day | 0.66 | 0.443 | 0.20-2.19 | 0.6878 | 2.08 | 0.231 | 0.57-7.65 | 0.5488 | NA | NA | NA | NA |
|  |  |  |  |  |  |  |  |  |  |  |  |  |  |
| Clean clothes | Every day | ref |  |  |  | ref |  |  |  | ref |  |  |  |
|  | Not every day | 0.44 | 0.320 | 0.07-2.64 | 0.4032 | NA | NA | NA | NA | 13.14 | 0.003 | 3.31-52.15 | 0.0001 |
|  |  |  |  |  |  |  |  |  |  |  |  |  |  |
| Clothes ironed | Never | ref |  |  |  | ref |  |  |  | ref |  |  |  |
|  | Sometimes | 0.93 | 0.554 | 0.73-1.20 |  | 0.96 | 0.755 | 0.72-1.29 |  | 1.28 | 0.148 | 0.90-1.84 |  |
|  | Always | 0.23 | 0.030 | 0.06-0.83 | 0.0607 | NA | NA | NA | NA | 2.10 | 0.285 | 0.47-9.39 | 0.3031 |
|  |  |  |  |  |  |  |  |  |  |  |  |  |  |
| Handwashing area in compound | No | ref |  |  |  | ref |  |  |  | ref |  |  |  |
|  | Yes | 0.77 | 0.079 | 0.57-1.04 | 0.1167 | 0.89 | 0.524 | 0.58-1.35 | 0.4503 | 0.83 | 0.277 | 0.58-1.19 | 0.3878 |
|  |  |  |  |  |  |  |  |  |  |  |  |  |  |
| Open fire in compound | No | ref |  |  |  | ref |  |  |  | ref |  |  |  |
|  | Yes | 1.59 | 0.015 | 1.12-2.26 | 0.0041 | 1.26 | 0.051 | 1.00-1.58 | 0.1434 | 1.32 | 0.386 | 0.66-2.65 | 0.1720 |
|  |  |  |  |  |  |  |  |  |  |  |  |  |  |
| Previous skin infection | None | ref |  |  |  | ref |  |  |  | ref |  |  |  |
|  | One | 3.14 | <0.001 | 2.12-4.65 |  | 1.95 | 0.002 | 1.40-2.72 |  | 2.41 | 0.002 | 1.57-3.71 |  |
|  | More than one | 4.86 | <0.001 | 2.83-8.33 | <0.0001 | 2.37 | 0.003 | 1.47-3.82 | <0.0001 | 2.25 | 0.040 | 1.05-4.82 | 0.0001 |
|  |  |  |  |  |  |  |  |  |  |  |  |  |  |
| History of burn | No | ref |  |  |  | ref |  |  |  | ref |  |  |  |
|  | Yes | 0.68 | 0.189 | 0.37-1.26 | 0.1878 | 1.01 | 0.959 | 0.63-1.63 | 0.9646 | 0.65 | 0.108 | 0.38-1.13 | 0.2182 |
|  |  |  |  |  |  |  |  |  |  |  |  |  |  |
| History of malnutrition | No | ref |  |  |  | ref |  |  |  | ref |  |  |  |
|  | Yes | 0.93 | 0.729 | 0.58-1.50 | 0.8194 | 0.71 | 0.339 | 0.33-1.54 | 0.2452 | 1.62 | 0.287 | 0.61-4.32 | 0.1592 |
|  |  |  |  |  |  |  |  |  |  |  |  |  |  |
| History of nutritional supplementation | No | ref |  |  |  | ref |  |  |  | ref |  |  |  |
|  | Yes | 0.33 | 0.243 | 0.04-2.50 | 0.1389 | 0.73 | 0.640 | 0.16-3.24 | 0.6038 | 0.59 | 0.404 | 0.15-2.33 | 0.4679 |

All values were corrected for cluster sampling design. OR = odds ratio; ref = reference category used; NA = regression analysis not possible due to too few participants; *significant at p<0.05; **significant at p<0.001; †Likelihood ratio test for inclusion of the variable in the model
