# Supplementary material for: High burden and seasonal variation of paediatric scabies and pyoderma prevalence in The Gambia: A cross-sectional study
Source: PLoS Negl Trop Dis. 2019 Oct 14;13(10):e0007801. doi: 10.1371/journal.pntd.0007801 (PMC6812840; doi:10.1371/journal.pntd.0007801)
Supplement: S5 Table — (DOCX) [file pntd.0007801.s010.docx]

|  | **Scabies** | | | **Pyoderma** | | | **Fungal** | | |
| --- | --- | --- | --- | --- | --- | --- | --- | --- | --- |
| **Model** | **Pseudo-R^2^** | **AIC*** | **LR test†** | **Pseudo-R^2^** | **AIC*** | **LR test†** | **Pseudo-R^2^** | **AIC*** | **LR test†** |
| Simple model | 0.0075 | 1262.503 | - | 0.0292 | 1303.954 | - | 0.0352 | 896.4075 | - |
| Step 1 | 0.0356 | 1225.17 | <0.0001 | 0.0396 | 1291.286 | 0.0001 | 0.0528 | 882.0818 | 0.0001 |
| Step 2 | 0.0538 | 1204.214 | <0.0001 | 0.0460 | 1284.711 | 0.0050 | 0.0685 | 869.4716 | 0.0001 |
| Step 3 | 0.0595 | 1199.045 | 0.0076 | 0.0508 | 1280.004 | 0.0171 | 0.0726 | 867.6772 | 0.0476 |
| Step 4 | 0.0638 | 1195.703 | 0.0203 | 0.0534 | 1278.547 | 0.0229 | 0.0753 | 867.1711 | 0.0947 |
| Step 5 | 0.0667 | 1193.965 | 0.0530 | 0.0580 | 1274.377 | 0.0434 | 0.0779 | 866.7833 | 0.1254 |
| Step 6 | 0.0692 | 1192.594 | 0.0926 | 0.0601 | 1273.316 | 0.1241 | 0.0807 | 865.9998 | 0.1487 |
| Step 7 | 0.0715 | 1191.592 | 0.0807 | 0.0624 | 1268.976 | 0.1739 | 0.0841 | 864.9258 | 0.0837 |
| Step 8 | 0.0738 | 1190.768 | 0.0926 |  |  |  | 0.0869 | 864.1161 | 0.1434 |
| Step 9 | 0.0761 | 1189.847 | 0.0887 |  |  |  |  |  |  |
| Step 10 | 0.0780 | 1189.15 | 0.1093 |  |  |  |  |  |  |
| Step 11 | 0.0798 | 1188.854 | 0.1282 |  |  |  |  |  |  |

Factor levels or variables were added to the simple model in a stepwise fashion including the next most significant, until the likelihood ratio test cut-off of >0.2 was reached. Sex and age category were included in the simple model and all subsequent steps. *Akaike information criterion; †Likelihood ratio test for model compared to previous step.
