# Supplementary material for: High burden and seasonal variation of paediatric scabies and pyoderma prevalence in The Gambia: A cross-sectional study
Source: PLoS Negl Trop Dis. 2019 Oct 14;13(10):e0007801. doi: 10.1371/journal.pntd.0007801 (PMC6812840; doi:10.1371/journal.pntd.0007801)
Supplement: S6 Table — (DOCX) [file pntd.0007801.s011.docx]

|  | **Scabies** | | | **Pyoderma** | | | **Fungal** | | |
| --- | --- | --- | --- | --- | --- | --- | --- | --- | --- |
| **Model** | **Pseudo-R^2^** | **AIC*** | **LR test†** | **Pseudo-R^2^** | **AIC*** | **LR test†** | **Pseudo-R^2^** | **AIC*** | **LR test†** |
| Global model | 0.0866 | 1209.772 | - | 0.0694 | 1293.344 | - | 0.1014 | 880.4532 | - |
| Step 1 | 0.0865 | 1207.813 | 0.8405 | 0.0694 | 1291.344 | 0.9546 | 0.1014 | 878.4744 | 0.8886 |
| Step 2 | 0.0865 | 1205.862 | 0.8248 | 0.0693 | 1289.345 | 0.9103 | 0.1014 | 876.4775 | 0.8626 |
| Step 3 | 0.0864 | 1204.305 | 0.7822 | 0.0693 | 1287.347 | 0.9081 | 0.1006 | 879.3588 | 0.8310 |
| Step 4 | 0.0863 | 1202.416 | 0.7388 | 0.0688 | 1286.043 | 0.8096 | 0.1005 | 877.4963 | 0.8022 |
| Step 5 | 0.0862 | 1200.566 | 0.7022 | 0.0688 | 1284.122 | 0.7288 | 0.1000 | 875.9853 | 0.5190 |
| Step 6 | 0.086 | 1198.757 | 0.6615 | 0.0686 | 1282.277 | 0.6985 | 0.0992 | 874.7197 | 0.4525 |
| Step 7 | 0.0855 | 1197.373 | 0.4236 | 0.0684 | 1280.591 | 0.6089 | 0.0986 | 873.2047 | 0.4674 |
| Step 8 | 0.0849 | 1196.424 | 0.3718 | 0.0683 | 1278.799 | 0.5958 | 0.0979 | 871.8654 | 0.4315 |
| Step 9 | 0.0843 | 1195.216 | 0.373 | 0.0680 | 1277.121 | 0.5951 | 0.0970 | 870.6887 | 0.3588 |
| Step 10 | 0.0836 | 1194.06 | 0.3654 | 0.0677 | 1275.505 | 0.5213 | 0.0962 | 869.3877 | 0.3587 |
| Step 11 | 0.083 | 1192.889 | 0.3647 | 0.0674 | 1273.987 | 0.4817 | 0.0959 | 867.6681 | 0.3328 |
| Step 12 | 0.0824 | 1191.689 | 0.3799 | 0.0671 | 1272.358 | 0.5104 | 0.0949 | 866.5976 | 0.3485 |
| Step 13 | 0.0817 | 1190.48 | 0.3729 | 0.0664 | 1271.196 | 0.4286 | 0.0937 | 865.7386 | 0.3021 |
| Step 14 | 0.0808 | 1189.585 | 0.2769 | 0.0656 | 1270.278 | 0.3858 | 0.0922 | 865.1465 | 0.2384 |
| Step 15 | 0.0798 | 1188.854 | 0.2635 | 0.0649 | 1269.218 | 0.3603 | 0.0905 | 864.8105 | 0.2004 |
| Step 16 |  |  |  | 0.0639 | 1268.596 | 0.2420 | 0.0887 | 864.5341 | 0.2078 |
| Step 17 |  |  |  | 0.0624 | 1268.976 | 0.2077 | 0.0869 | 864.1161 | 0.2117 |

Factor levels or variables were removed from the global model in a stepwise fashion removing the next least significant, until the likelihood ratio test cut-off of <0.2 was reached. Sex and age category were included in all models and steps. *Akaike information criterion; †Likelihood ratio test for model compared to previous step.
