# Supplementary material for: High burden and seasonal variation of paediatric scabies and pyoderma prevalence in The Gambia: A cross-sectional study
Source: PLoS Negl Trop Dis. 2019 Oct 14;13(10):e0007801. doi: 10.1371/journal.pntd.0007801 (PMC6812840; doi:10.1371/journal.pntd.0007801)
Supplement: S7 Table — (DOCX) [file pntd.0007801.s012.docx]

|  |  | **Swab site** | | | | |
| --- | --- | --- | --- | --- | --- | --- |
|  |  | **All** | **Head** | **Trunk** | **Upper limbs** | **Lower limbs** |
|  |  | n (%) | n (%) | n (%) | n (%) | n (%) |
|  |  |  |  |  |  |  |
| *S. aureus* positive | No | 48 (19.2) | 21 (16.8) | 3 (14.3) | 4 (18.2) | 20 (24.4) |
|  | Yes | 202 (80.8) | 104 (83.2) | 18 (85.7) | 18 (81.8) | 62 (75.6) |
|  |  |  |  |  |  |  |
| GAS positive | No | 123 (49.2) | 85 (68.0) | 8 (38.1) | 9 (40.9) | 21 (25.6) |
|  | Yes | 127 (50.8) | 40 (32.0)* | 13 (61.9) | 13 (59.1) | 61 (74.4)* |
|  |  |  |  |  |  |  |
| Mixed growth | No | 146 (58.4) | 91 (72.8) | 10 (47.6) | 10 (45.5) | 35 (42.7) |
|  | Yes | 104 (41.6) | 34 (27.2)* | 11 (52.4) | 12 (54.6) | 47 (57.3)* |
|  |  |  |  |  |  |  |
| No growth | No | 225 (90.0) | 110 (88.0) | 20 (95.2) | 19 (86.4) | 76 (92.7) |
|  | Yes | 25 (10.0) | 15 (12.0) | 1 (4.8) | 3 (13.6) | 6 (7.3) |
|  |  |  |  |  |  |  |
| Total |  | 250 (100.0) | 125 (50.5) | 21 (8.4) | 22 (8.8) | 82 (32.8) |

Chi-squared test for significance performed for each growth at each swab site. *p<0.001
