# Supplementary material for: High burden and seasonal variation of paediatric scabies and pyoderma prevalence in The Gambia: A cross-sectional study
Source: PLoS Negl Trop Dis. 2019 Oct 14;13(10):e0007801. doi: 10.1371/journal.pntd.0007801 (PMC6812840; doi:10.1371/journal.pntd.0007801)
Supplement: S8 Table — (DOCX) [file pntd.0007801.s013.docx]

|  |  | **Scabies** | | | **Pyoderma** | | | **Fungal** | | |
| --- | --- | --- | --- | --- | --- | --- | --- | --- | --- | --- |
|  |  | **PR** | **p value** | **95% CI** | **PR** | **p value** | **95% CI** | **PR** | **p value** | **95% CI** |
| Full sample (n=1441) | |  |  |  |  |  |  |  |  |  |
| Start of rains | Before (n=575) | ref |  |  | ref |  |  |  |  |  |
|  | After (n=866) | 1.08† | 0.702 | 0.70-1.67 | 2.42† | 0.006* | 1.38-4.23 | 0.44† | <0.001** | 0.32-0.60 |
|  |  |  |  |  |  |  |  |  |  |  |
| First / last cluster only (n=207) | |  |  |  |  |  |  |  |  |  |
| Start of rains | Before (n=101) | ref |  |  |  |  |  |  |  |  |
|  | After (n=106) | 1.59 | 0.370 | 0.58-4.37 | 2.74 | 0.014* | 1.23-6.12 | 0.60 | 0.363 | 0.19-1.82 |
|  |  |  |  |  |  |  |  |  |  |  |
|  |  | ***S. aureus* positive pyoderma** | | | **GAS positive pyoderma** | | |  |  |  |
|  |  | **PR** | **p value** | **95% CI** | **PR** | **p value** | **95% CI** |  |  |  |
| All pyoderma (n=250) | |  |  |  |  |  |  |  |  |  |
| Start of rains | Before (n=50) | ref |  |  | ref |  |  |  |  |  |
|  | After (n=200) | 1.07† | 0.571 | 0.81-1.42 | 0.99† | 0.913 | 0.75-1.30 |  |  |  |

ref = reference category used; PR = prevalence ratio; †Adjusted for sex, age group, tribe, household size and mother’s education; corrected for cluster sampling design; *significant at p<0.05; **significant at p<0.001
