# Supplementary material for: High burden and seasonal variation of paediatric scabies and pyoderma prevalence in The Gambia: A cross-sectional study
Source: PLoS Negl Trop Dis. 2019 Oct 14;13(10):e0007801. doi: 10.1371/journal.pntd.0007801 (PMC6812840; doi:10.1371/journal.pntd.0007801)
Supplement: S9 Table — (DOCX) [file pntd.0007801.s014.docx]

|  | **Prevalence^**  **(%)** | **Sensitivity**  **(%)** | **Specificity**  **(%)** | **Kappa statistic*** |
| --- | --- | --- | --- | --- |
| No skin problem | 44.4 | 98.2 | 94.2 | 0.92 |
| Non-infected scabies | 19.4 | 83.3 | 97.0 | 0.82 |
| Pyoderma | 28.2 | 97.1 | 96.6 | 0.92 |
| Infected scabies | 6.5 | 62.5 | 98.3 | 0.65 |
| Scabies and pyoderma (any combination)† | 12.9 | 81.3 | 97.2 | 0.78 |
| Fungal infection | 4.8 | 66.7 | 95.8 | 0.50 |

Study undertaken in a subset of 124 participants. ^As determined by the physician; *Kappa ranges from -1 to 1, generally values greater than >0.75 - excellent agreement, 0.4 to 0.75 - fair to good agreement, 0.4 indicate moderate or poor agreement; †Diagnosis of non-infected scabies and pyoderma, or infected scabies
